# Efficacy of Glucose-Insulin-Potassium Infusion on Left Ventricular Performance in Type II Diabetic Patients Undergoing Elective Coronary Artery Bypass Graft.Dy

**Published:** 2010

**Authors:** Seied Mostafa Seied-Hosseini, Ali Pourmoghadas, Omid Aghadavoudi, Masoud Amini, Mohsen Mirmohammad-Sadeghi, Allahyar Golabchi, Behnood Hedayatpour, Elham Haratian, Narges Ghaem-Maghami, Laleh Khanoom Sharegh

**Affiliations:** 1 Resident of Cardiology, Department of Cardiology, School of Medicine, Isfahan University of Medical Sciences, Isfahan, Iran; 2 Associate Professor of Cardiology, Department of Cardiology, School of Medicine, Isfahan University of Medical Sciences, Isfahan, Iran; 3 Associate Professor of Anesthesiology, Department of Anesthesiology, School of Medicine, Isfahan University of Medical Sciences, Isfahan, Iran; 4 Professor of Endocrinology, Endocrine and Metabolism Research center, Isfahan University of Medical Sciences, Isfahan, Iran; 5 Assistant professor of Surgery, Department of Heart Surgery, School of Medicine, Isfahan University of Medical Sciences, Isfahan, Iran; 6 School of Medicine, Isfahan University of Medical Sciences, Isfahan, Iran; 7 Isfahan University of Medical Sciences, Isfahan, Iran

**Keywords:** Cardiovascular surgery, Glucose-Insulin-Potassium, Cardiac troponin

## Abstract

**BACKGROUND:**

Glucose-insulin-potassium (GIK) may improve cardiovascular performance after coronary artery bypass graft surgery (CABG). Our study investigated whether an infusion of GIK during elective CABG surgery in type II diabetic patient improved left ventricular performance.

**METHODS:**

We measured left ventricular ejection fraction and troponin (Tn), a myofibrillar structural protein. In this research, after ethics committee approval, 50 patients with type 2 diabetes mellitus (DM) were enrolled into a randomized simple sampling, prospective, double-blind clinical trial study. In the case group, 500 cc dextrose water 5% plus 80 IU regular insulin and 40 mEq KCL were infused at the rate of 30 cc/hr. Patients in control group received 5% dextrose solution at the rate of 30cc/hr. Venous blood samples were taken before induction of anesthesia, after removal of the aortic clamp and before discharging from hospital. The Mann-Whitney-test was used to test for differences in troponin concentration between the groups. Fisher's exact test was used to determine whether there was a difference in the proportion of patients with a low ejection fraction (<45%) in the case group compared with that in the control group. Changes in potassium and glucose concentrations over time within the groups were examined by ANOVA and paired t-tests. P < 0.05 was regarded as significant level for all tests.

**RESULTS:**

In this study, 50 patients with type 2 DM were evaluated in case and control groups. The mean age ± SD in the case group was 57.7 ± 9.9 years and in the other group was 61.2 ± 8.4 years. The groups were well-matched for age, sex and number of bypass grafts. Randomization did not give an equal distribution of male and female patients. There wasn't any significant difference in ejection fraction between the case and control groups before and after CABG (P > 0.05). Troponin concentration in the case group was 3.3 ± 5.0 and in the control group was 3.9 ± 5.1. There was no significant difference in Tn between the two groups before and after CABG (P > 0.05). There was not any significant difference in hospitalization time between the two groups.

**CONCLUSION:**

The results suggested that GIK can't improve left ventricular performance in routine CABG surgery.

## Introduction

An increasing number of diabetic patients with coronary artery disease have been referred to surgical treatment, considering that the myocardial revascularization surgery is the treatment of choice for the majority of these patients.^1^, ^2^ Diabetes mellitus may be considered as an independent factor for both mortality and complications after the myocardial revascularization surgery and the motivation to reduce these episodes have recently renewed the interest around the investigation of glucose-insulin-potassium (GIK). Several experimental studies have evaluated the possible action mechanisms of GIK^3^–^7^, and the treatments of acute myocardial infarction in diabetic patients have produced convincing evidence of GIK benefits.^8^, ^9^ Some studies have shown a better hemodynamic performance using the GIK in postoperative diabetic patients undergone myocardial revascularization graft surgery .^10^, ^11^ The use of GIK in myocardial revascularization surgery has been introduced as a source of metabolic support to the ischemic myocardium since the 1960s,^12^ nevertheless, remained controversial. During hypoxia, the heart has limited oxidative reserve; and energy-rich phosphates are steadily depleted. In hypoxia, GIK may protect myocardial tissue by maintaining normal carbohydrate and fatty acid metabolism and thus, cell function. The effects of GIK and its influence on myocyte metabolism, especially during ischemia and reperfusion, are complex. The protective effect of GIK on the functional recovery of the heart has been investigated extensively.^13^–^16^ The different outcomes of GIK therapy on acute myocardial infarction type II diabetic patient, various protocols used, and different administered doses and various periods evaluated, make it difficult to analyze the significant impact of GIK usage. The patients with diabetes mellitus (DM) could drive a great benefit from the use of GIK, given the glycemic control, the decreased non-esterified fatty acid of plasma concentrations and the substrate intake to the myocardium at the ischemic trans-operative period are important factors in the patient's postoperative recovery. Our study investigated whether an infusion of GIK during elective coronary artery bypass graft (CABG) surgery in type II diabetic patients improved left ventricular performance.

## Methods

The patients were selected with simple sampling in this clinical trial study. It was a randomized, prospective study. Patients with type 2 DM with multivessel coronary artery disease admitted to the Shahid-Chamran hospital in Isfahan, Iran, from January 2007 to January 2010 were eligible to participate in this study. The DM diagnosis was made given the patients' clinical history treated with oral hypoglycemic agents or insulin and/or fasting hyperglycemia >126 mg/dL. After approval of ethics committee, we conducted a double-blind prospective randomized clinical trial study of 50 patients with type 2 DM who were scheduled for elective CABG. Exclusion criteria were the patients with chronic renal failure (with creatinine > 2), acute renal failure (urine output < 30 ml/hr * 3 times), hyperkalemia (K >5.5 mEq/L) and hepatic failure with unknown causes. The patients with other causes of heart surgery were excluded from the study. Assessment of ejection fraction was part of the routine preoperative evaluation. After getting informed consent, all patients enrolled into study were evaluated for ejection fraction by 2D echo, m-mode with vivid 3 echocardiography. The patients received either 500 cc dextrose water 5% plus 80 IU regular insulin and 40 mEq KCL at a rate of 30 cc/hr (case group) or an equivalent rate of 5% dextrose (control group), given into a central vein. The infusion started immediately after induction of anesthesia and continued for 12 hours after CABG surgery. Blood glucose was measured hourly with glucometer and insulin was given according to the tables 1 and 2.

**Table 1:** Schedule to set GIK infusion (case).^17^,^18^

Glycemic index (mg/dl)	Conduct of treatment
> 270	8 IU of human regular insulin in bolus + GIK increase to 6 ml/h GIK increase to 3 ml/h
201–270	No Change
126–200	GIK rate decrease to 6 ml/h
75–125	Stop GIK for 15 minutes repeating the blood sugar (BS) measurement every 15 minutes until BS reached >125 mg/dl.
<75	Then, reinitiate GIK infusion with a rate of 6 ml/h.

**Table 2:** Schedule for administration of insulin subcutaneously (control)

Glycemic index (mg/dl)	Conduct of treatment
80–250	No insulin
251–300	4 Units of regular human insulin, subcutaneously
300–350	6 Units of regular human insulin, subcutaneously
350–400	8 Units of regular human insulin, subcutaneously

Potassium was added to achieve a plasma concentration of 4-6 mmolper liter. Peripheral venous blood samples were collected for Tn analysis in the case and the control groups, 24 hours after CABG. The heart surgery complications such as atrial fibrillation and the death in the hospitalization period and 3 months later were recorded and compared between the two groups. Functional class was measured pre- and post- operatively (3 months later) according to NYHA criteria. The duration from intubation point to the extubation point in ICU called intubation time. The duration from clamping the ascending aorta to the removal of clamp called clamp time. Pump time was the time that patients were on cardiopulmonary pump.

### Blood sampling and laboratory analysis

Glucose and potassium plasma concentrations were measured pre-CABG, during CABG (trans-CABG) and on the 1^st^, 2^nd^, 4^th^, 6^th^, 8^th^, 12^th^, 18^th^, and 24^th^ hours, postoperatively. Blood glucoses were obtained at each hour until the 6^th^ hour, postoperatively and then, at each 2 hours until the 24^th^ hour. Blood samples were taken into tubes without anticoagulant and centrifuged at 2500g for 20min. The serum obtained was then stored at -70°C until analysis. Quantitative Tn analysis was performed using the Bayer Immuno-assay (Bayer, Leverkusen, Germany), a heterogeneous sandwich magnetic separation system using mouse monoclonal and goat polyclonal anti-Tn antibodies. The randomization was blinded to patients, surgeons, and cardiologists, who were directly involved in the accomplishment of the protocol and in the measurements, such as clinical, laboratory, and hemodynamic data. The participants were randomized into either the intervention or control groups. Only the anesthesiologist had access to the group information to which the patients was randomized.

### Statistical analysis

A power analysis showed that 25 patients in the case and control groups were required to detect a mean difference of 0.9 (SD 0.8)µgper liter in Tn concentration between the two groups at any time point with a power of 90% and a P value of 0.05. Non-parametric statistical tests were therefore used for (Tn) analysis. The Mann-Whitney test was used to test the differences in Tn concentration between the two groups at each time point. Changes in Tn concentration over time within the groups were assessed by Friedman analysis of variance (ANOVA) and the Wilcoxon signed ranks test. Fisher's exact test was used to determine whether there was a significant difference in the proportion of patients with a low ejection fraction (< 45%) in the case group compared with that in the control group. Other categorical data, including hypertension, hypercholesterolemia, preoperative myocardial infarction and gender ratio) were also assessed with Fisher's exact test. The Mann-Whitney test was used to determine whether Tn concentrations were greater in patients with low ejection fractions. The relationship between cross-clamp time and Tn concentration was evaluated by Spearman correlation analysis. Patient age, CABG time, aortic cross-clamp time and blood glucose and blood potassium concentrations were normally distributed and were assessed with parametric statistical tests. Differences in these variables between the case and control groups were assessed with t-test. Changes in potassium and glucose concentrations over time within the groups were examined by ANOVA and paired t-tests. P < 0.05 was regarded as significant level for all tests.

## Results

The groups were well-matched for age and number of bypass grafts (Table 3). One patient in group 1 (the case group) and two patients in group 2 (the control group) had four grafts, and four ones in group 1 and 11 patients in group 2 received two grafts. All other patients had three coronary artery grafts with at least one internal mammary artery graft.

**Table 3:** Patients’ characteristics. Values are presented as mean (SD).

	Case (GIK group )	Control (Non-GIK group)	P-value
Age (years)	57.7 (9.9)	61.2 (8.4)	0.18
Gender ratio (M:F)	9:16	14:6	0.04
Body mass Index (BMI)	28.7 (5.0)	28.0 (4.7)	0.62
Pump time (min)	99.0 (23.7)	99 (27.1)	0.6
clamp time (min)	59.5 (15.0)	61.4 (17.0)	0.68
Systolic BP	133.8 (18.3)	126.5 (24.1)	0.23
Cholesterol	190.2 (55.1)	190.9 (44.0)	0.96
Diabetes duration (years)	5.1 (5.2)	7.4 (6.2)	0.19
Ejection fraction	50.6 (8.1)	49.5 (12.4)	0.7
Intubation Time (minutes)	662.2 (208)	734.6 (287.5)	0.32
Death (in-hospital)	0	1	0.49

Randomization did not give an equal distribution of male and female patients. Mean ejection fraction before surgery in the case and control groups were 50.6% ± 8.1 and 49.5% ± 12.4, respectively (Table 3). Mean ejection fraction before discharge in the case and control groups were 44.1 ± 6.8 and 46.3 ± 14.9, respectively. There was not a significant difference in the mean ejection fraction between the two groups before and after CABG (P > 0.05, Table 4). There were not significant changes in ejection fraction (EF1-EF2) in the case and control groups after CABG (P > 0.05).

**Table 4:** Mean ejection fraction in both groups before and after CABG. Values are presented as mean (SD).

	Case group	Control group	P-value
EF before CABG	50.6 (8.1)	49.5 (12.4)	0.7
EF after CABG	44.1 (6.8)	46.3 (14.9)	0.5
EF1–EF2	−6.52 (6.9)	−4.13 (6.5)	0.2

Before operation, all patients were on beta-blockers and aspirin; the latter was stopped 5 days before surgery. A large number were also taking cholesterol-lowering medications (statins) and angiotensin-converting enzyme inhibitors.

### Tn concentration

Mean Tn concentrations in the case and control groups were 3.3 ± 5.0 and 3.9 ± 5.1, respectively. There was not a significant difference in Tn concentrations between the two groups after CABG (P > 0.05).

### Hospitalization

The mean hospitalization periods in the case and control groups were 7.4 ± 8.7 and 7.5 ± 8.8 days, respectively. There was not a significant difference in hospitalization periods between the two groups (P = 0.8). (Table 5)

**Table 5:** Mean hospitalization periods in both groups. Values are presented as mean (SD)

	Case group	Control group	P-value
Hospitalization	7.4 (8.7) days	7.5 (8.8) days	0.8

**Table 6:** Mean potassium and BS levels before, during and after the CABG in both groups.Values are presented as mean (SD)

	Case group	Control group	P-value
Potassium (before)	4.40 (0.5) meg/L	4.44 (0.48) meg/L	0.7
Potassium (during)	4.16 (0.4) meg/L	4.42 (0.49) meg/L	0.03
Potassium (after)	4.15 (0.6) meg/L	4.18 (0.42) meg/L	0.8
BS (before)	220.6 (105.4) meg/L	188.7 (72.2) meg/L	0.2
BS(during)	206.0 (47.4) meg/L	244.7 (73.2) meg/L	0.03
BS (after)	180.0 (56.1) meg/L	187.8 (58.5) meg/L	0.6

### Functional Class (FC)’

Two patient in the case group and no patient in the control group had poor FC (class IV), 13 in the case group and 9 patients in the control group had fair FC (class III), and 9 in the case group and 12 patients in the other group had good FC (class II). All other patients had excellent FC (class I) before CABG (Table 7).

**Table 7:** Functional class classification in both groups before CABG.

Functional Class	Case group	Control group
excellent(I)	1	3
good(II)	9	12
fair(III)	13	9
Poor (IV)	2	0

There were significant changes in FC (FC2-FC1) within the case and control groups after and before CABG (P = 0.001, Table 7 and 8).

**Table 8:** Functional class classification in both groups 3 months after CABG.

Functional Class	Case group	Control group
excellent(I)good(II)Flair	21	17
(III)	3	7
Poor(IV)	1	
	0	0

There weren't any significant changes in atrial fibrillation between the case and control groups after CABG (P = 0.28, Table 9).

**Table 9:** Atrial fibrillation (AF) in both groups after CABG.

AF	Case group	Control group	P-Value
Yes	1 (4%)	3 (12.5%)	0.28

## Discussion

Perioperative myocardial infarction is a clinically recognizable form of myocardial damage and has a side effect.^17^ The infusion of GIK solutions as a metabolic support to myocardium in the perioperative period of myocardial revascularization surgery has been recommended many years ago, but there is no consensus regarding its benefits. Interest has also grown around the potential usage of GIK infusions due to the increasing number of diabetic patients submitted to surgical treatment and the search for better outcomes. The GIK effects in experimental studies have shown its capacity to preserve the contractile function after an ischemic period and consequently, a better hemodynamic performance of the heart is expected.^3^–^7^ In this research, we studied whether an infusion of GIK during elective CABG surgery in type II diabetic patients improved left ventricular performance. To quantify the amount of left ventricular performance perioperatively, we used Tn concentration, ejection fraction and functional class.^17^ The maximum value of Tn can be used to quantify the extent of myocardial cell death.^17^, ^18^ Tn is accepted as a marker of perioperative risk stratification in cardiac risk patients undergoing non-cardiac surgery^19^ and in patients undergoing CABG, because its concentration is related to the amount of irreversible cell injury. Higher perioperative concentrations are associated with more postoperative complications.^20^ Ejection fraction and functional class are related to the amount of individual capacity and functional capacity of the heart. It has also been used to indicate underlying cardiac injury in intensive care patients^21^, ^22^ and to examine the benefits of different solutions and temperatures of cardioplegia.^23^ The heart is most likely to sustain ischemic injury when the aortic cross-clamp is applied. This is supported by our finding of a correlation between cross-clamp time and peak Tn concentration.

In this study, despite the theoretical protective effects of GIK listed above and the increase in functional class, no cardioprotective effect of GIK was observed. We did not find a significant difference in ejection fraction between the two groups. Maybe, our results supported the findings of other studies,^13^ but there was no difference between GIK and non-GIK-treated patients. Before concluding that GIK confers no protective effect on the myocardium during CABG surgery, other possible reasons for our negative findings should be considered. We used a high dose of GIK, as recommended in other studies,^24^, ^25^ but continued this only 12 hours after surgery. It might be of more benefit if started before surgery and continued for 24-48 hours after surgery, as suggested by other studies. Some researchers examined the effect of GIK on complications of mitral valve replacement and demonstrated increased myocardial glycogen content after an infusion of GIK, 12 hours before the operation.^26^ This reduced complications such as hypothermia and arrhythmia after surgery. Lazar and colleagues^27^ found better cardiac performance and faster recovery from urgent CABG in patients with unstable angina given GIK infusion for 12 hours after operation. These patients had greater cardiac output, a significantly lower incidence of atrial fibrillation (13.3 vs. 53.3%, P = 0.02) and a shorter stay in intensive care. GIK might be beneficial in patients with decreased cardiac reserve, poor left ventricular function and/or cardiogenic shock. Patients with a high risk of death may be more likely to benefit from metabolic therapy.^28^ Patients with insulin-dependent diabetes might benefit particularly from GIK, as shown in the DIGAMI study for patients with myocardial infarction.^29^ A recent study by Lazar and colleagues found better cardiac performance and faster recovery after CABG in a group of 40 diabetic patients.^30^ Postoperative GIK infusion for 48 hours was of benefit in patients with refractory cardiac failure after hypothermic cardiac arrest for CABG.^31^ Other authors have been using cardiac index (CI) to evaluate the benefits from GIK in myocardial revascularization surgery. A randomized clinical trial published in 2000 compared the diabetic patients undergone myocardial revascularization surgery using GIK (n=20) or conventional treatment with insulin subcutaneously (n=20). CI has been shown to increase in the intervention group (CI at the 18^th^ hour postoperative: 2.88 ± 0.50 vs. 2.2 ± 0.39 L/min/m^2^ in the case and control groups, respectively, 95% CI 0.38-0.97, P < 0.0001).^10^ Szabó et al^11^ have studied the diabetic patient undergone to myocardial revascularization surgery and randomized to the case group with high doses of insulin (n=10) or conventional treatment (n=10). In the case group there was a significant increase in the CI (2.3 ± 0.1 vs. 2.9 ± 0.2 L/min/m^2^, P = 0.017). An essay published in 2000 evaluated 45 patients undergone to myocardial revascularization surgery without cardiopulmonary pump and randomized to GIK or saline solution. It was not found any difference between the groups regarding CI and SvO_2_ (mixed venous oxygen saturation) as well as in the enzymatic variables CPK-MB and troponin I.^12^ The Insulin Cardioplegia Trial^13^ evaluated the use of 10-IU insulin-fortified cardioplegia vs. placebo in high-risk patients undergone myocardial revascularization surgery due to unstable angina. A total of 1127 patients were randomized. There was no significant difference regarding mortality rate and the event of enzymatic infarction and/or low cardiac output syndrome. This study was criticized by not considering some existing evidences, such as low insulin dose administered in a single moment and not maintained the intervention in the postoperative period.^32^ A meta-analysis published in 2004, which included 11 studies evaluating the GIK administered in the CABG postoperatively or in the valve replacement relating to 468 patients, estimated an 11.4% increment in the CI of patients who received GIK infusion with a decrease in the atrial fibrillation in the postoperative period.

### Limitations

It is possible that our study was too small and the groups were too poorly matched to reveal the benefit of GIK. The different proportions of male and female patients in the case and control groups are unlikely to have influenced the results, but this possibility cannot be excluded. The possible influence of the ejection fraction was analyzed retrospectively. The importance of the ejection fraction as a confounding factor is uncertain as there was no significant difference in Tn concentration at any time point between patients with low and those with normal ejection fractions. A problem with high glucose infusion is hyperglycemia during CABG, which could damage the brain. Hyperglycemia may increase ischemic brain injury. A confounding factor is that high glucose concentrations may indicate severe head injury, as hyperglycemia is a general stress response.^29^ However, the blood glucose concentration should be maintained within the normal range. Measuring glucose and potassium concentrations regularly is necessary.

## Conclusion

We did not find that the use of GIK infusion during surgery to be useful in myocardial cell damage and ejection fraction associated with cardiac surgery and CABG. So, routine GIK infusions cannot be recommended until further studies to be performed that demonstrate the value of this practice and determine which patients are most likely to benefit.
